# Comparing isolated-check visual evoked potential, pattern visual evoked potential, and standard automated perimetry in dysthyroid optic neuropathy eyes

**DOI:** 10.1038/s41433-020-01274-3

**Published:** 2020-11-13

**Authors:** Xin Qi, Boding Tong, Weikun Hu, Ban Luo

**Affiliations:** 1 Department of Ophthalmology, the Second Xiangya Hospital, Central South University, Changsha, 410011 Hunan China; 2 Department of Ophthalmology, Tongji Hospital, Tongji Medical College, Huazhong University of Science and Technology, Wuhan, 430030 Hubei China

**Keywords:** Optic nerve diseases, Autoimmune diseases

## Abstract

**Objective:**

To determine the diagnostic ability of isolated-check visual evoked potential (icVEP), pattern visual evoked potential (pVEP), and standard automated perimetry (SAP) between dysthyroid optic neuropathy (DON) and thyroid-associated ophthalmopathy (TAO) without DON (non-DON).

**Methods:**

This is a case-control study, 49 bilateral patients (26 DON and 23 non-DON) were included. icVEP, pVEP, and SAP were conducted in all the subjects, icVEP parameters compared were signal-to-noise ratios (SNRs) under 8, 16, and 32% depth of modulation (DOM). pVEP parameters compared were amplitude and latency. SAP parameters were mean deviation (MD) and pattern standard deviation (PSD). The area under the receiver operating characteristic (ROC) curve (AUC), net reclassification index (NRI), integrated discrimination index (IDI), and decision curve analysis (DCA) were applied for analysis.

**Results:**

In icVEP, values of SNR in DON were significantly smaller than non-DON (*p* < 0.05). In pVEP, P100 latent time in DON was significantly larger than non-DON (*p* = 0.0026). In SAP, value of PSD in DON was larger than non-DON (*p* = 0.0006), and value of MD in DON was smaller (*p* = 0.0007). AUC, NRI, and IDI among the three tests were not significantly different. DCA showed that SNR of icVEP under 8% DOM was the farthest from the two extreme curves.

**Conclusions:**

icVEP, pVEP, and SAP have equal diagnostic capabilities to discern between DON and non-DON. In addition, icVEP may represent a significant ancillary diagnostic approach to DON detection, with more clinical benefit.

## Introduction

Dysthyroid optic neuropathy (DON) is a sight-threatening condition that occurs due to impaired optic nerve function secondary to thyroid-associated ophthalmopathy (TAO) [1, 2]. It has an estimated incidence of 3–8.6% [2–4]. Without accurate diagnosis and timely intervention, there is a risk of sight loss. Although the European Group on Graves’ Orbitopathy [5] (EUGOGO) established a standard clinical criteria assessment, there are still controversy and challenges regarding the diagnostic features of DON, and recognition of it might be delayed or even atypical [2, 3]. These make a “gold standard” or objective and sensitive clinical test essential for early diagnosis to reduce morbidity and improve prognosis.

Ancillary tests, including visual evoked potentials (VEPs) and standard automated perimetry (SAP), have been applied to objectively assess the presence, predict the development and estimate the severity of DON [6]. The majority of eyes with DON develop a central or paracentral scotoma and peripheral breakout patterns [3, 7]; thus, visual field testing, such as SAP, could accurately detect this disease. VEPs are signals obtained from electroencephalographic activities [8] and can examine the functional integrity of the visual pathway, including the retina, optic nerve, optic radiation, and occipital cortex [9], making them helpful for detecting and monitoring DON and possibly being more sensitive than kinetic perimetry [3, 7]. Pattern VEP (pVEP) is widely used for DON screening; however, isolated-check VEP (icVEP) was designed based on known physiological properties of visual neurons, and it is assumed to be more specific to the early stage of optic neuropathy [10]. Nevertheless, little information is available regarding the potential diagnostic ability and clinical utility of the above tests. The purpose of this study was to compare the diagnostic ability of these three unique tests (icVEP, pVEP, and SAP) in a DON group compared to a TAO without DON (abbreviated as non-DON) group, aiming to provide useful information about the diagnostic and follow-up utility of these tests for DON patients in clinical practice.

## Materials and methods

### Subjects

This is a case-control study, 49 bilateral patients (26 DON and 23 non-DON) were included. The study was approved by the Ethics Committee of the Tongji Hospital of Huazhong University of Science and Technology. It was carried out with rules and principles of the Declaration of Helsinki. All subjects were recruited from Tongji Hospital and signed informed consent forms prior to participation.

The diagnosis of TAO and the severity classification followed the EUGOGO criteria [5]. The inclusion criteria for TAO patient: (1) age >18 years, (2) clear refractive media allowing sufficient image quality, and (3) no treatment with systemic glucocorticoids for at least 3 months prior to the study. The exclusion criteria: (1) any systemic diseases other than thyroid disorders, (2) any history of ocular surgery or trauma, (3) any ophthalmic diseases other than TAO, and (4) any neurological abnormalities that could account for visual field (VF) changes. DON was diagnosed based on the presence of two or more of the following clinical findings: relative afferent pupillary defect (RAPD) when unilaterally affected, colour vision defect, decreased visual acuity, papilledema, VF defect, and abnormal pVEP. Both eyes of the enroled subjects were diagnosed with TAO with or without DON.

### Ophthalmic and systemic examination

Each subject underwent comprehensive ophthalmic examinations, including best-corrected central visual acuity (BCVA), refraction, colour vision test, intraocular pressure (IOP) measurement, slit-lamp microscopy, ophthalmoscopy, RAPD evaluation, and exophthalmometry (Hertel exophthalmometer). The clinical activity of TAO was graded according to the Clinical Activity Score (CAS) [11]. pVEP test, icVEP test, and SAP were performed on different days considering the effect of fatigue. Both eyes of the subjects were consecutively tested under a natural pupil size, and one of two eyes was randomly selected for statistical analysis. All subjects underwent more than one SAP test considering learning effects, and only the results of the second reliable SAP test were used in analysis. Demographic information was collected for all, including age, sex and medical history, such as hyperthyroidism, radioiodine (I131) therapy history, and smoking history.

### icVEP

All eyes underwent icVEP testing (MKWHAMD, CN-V1.4, Huzhou Medconova Medical Technology Co. LTD, Huzhou, China) without pupil dilation (diameters ranged from 2.5 to 4.0 mm). We used swept-parameter stimuli with five increasing levels of the depth of modulation (DOM) of the check luminance: 2, 4, 8, 16, and 32% at duration of 2 s each with the signal-to-noise ratio (SNR) calculated. The first two stimuli (2 and 4%) were included for adaptation purposes. An auditory signal and a fixation cross preceded the start of a run. The spatial pattern was a 10 × 10 array of isolated checks (30′ check width). It subtended 10° at the viewing distance of 70 cm. The luminance of the display’s static background was 50 cd/m^2^. Each pattern was presented in appearance–disappearance mode with Weber contrast modulated by the temporal sinusoid. The resultant icVEP is the vector mean of eight runs. The SNR is a measure of estimated VEP response magnitude relative to the noise (response variability), it is defined as the ratio of the vector-mean amplitude (magnitude) of the fundamental component to the radius of the noise circle: SNR = M_mean_/r.

### pVEP

pVEP was performed using the Neucodia visual electrophysiological diagnostic system (Huzhou Medconova Medical Technology, Inc.). Data were obtained using a black-and-white reversing checkboard stimulus (80% contrast, 85 cd/m^2^ luminance) subtending 50’ of arc at the subject’s eye at 50 cm. All recordings were transient and used a stimulus rate of 2 Hz. For pVEP, a check size of 50’ was chosen because it is large enough to be seen by a subject with early lens opacities and yet still stimulates the central pathways [12]. The delayed P100 latent time or/and reducted P100 amplitude were considered abnormal, and were recorded for further analysis.

### SAP

SAP was performed using the Humphrey Automated Field Analyser 740i (Carl Zeiss Meditec, Inc.) with the 24–2 standard procedure using standard test parameters after obtained refractive correction. The mean deviation (MD) < −1.0 dB and pattern standard deviation (PSD) significant at *p* < 0.05 were considered abnormal and were obtained for further analysis.

### Statistical analysis

All the analyses were performed using Empower (R Version 2.0). Kolmogorov–Smirnov test was used to assess the normality of the distribution of continuous data. The differences between non-normally distributed continuous variables were compared using Mann–Whitney *U*-test, and categorical variables were compared using the Chi-square test or Fisher’s exact test. The receiver operating characteristic (ROC) curves were constructed using bootstrap resampling (times = 500) and were used to determine the discriminatory capabilities of the tests for differentiating between DON and non-DON eyes. The area under the ROC curve (AUC), the net reclassification index (NRI), and the integrated discrimination index (IDI) were evaluated. When comparing two tests, a value of NRI or IDI greater than 0 demonstrates that the latter is better than the former, and vice versa. AUCs were compared using MedCalc (MedCalc Software 12.7.8.0, Mariakerke, Belgium). Decision curve analysis (DCA) was used to assess the clinical utility of different tests for decision making. *p* values < 0.05 were considered statistically significant.

## Results

### Demographic and clinical characteristics of the subjects

A total of 23 non-DON subjects (23 eyes) and 26 DON subjects (26 eyes) were enroled in this study. The demographic and clinical characteristics of all subjects are summarised in Table 1. Except for a slight age imbalance between groups, there were no significant differences between the non-DON and DON groups in sex distribution, right/left eye selected, IOP, refraction, degree of exophthalmos, active phase, current thyroid condition, duration of the disease, history of I131 treatments, or history of smoking. There were significant differences between the two groups in CAS, as well as visual functions, such as BCVA, history of decreased VA, papilledema, VF defect, and abnormal pVEP, as expected. However, colour vision and RAPD between the two showed no differences.

**Table 1:** Demographic characteristics of the study subjects.

	Non-DON (*n* = 23)	DON (*n* = 26)	*p* value
Age (year)	47.74 ± 8.94	53.38 ± 5.76	**0.014**^a^
Sex (male/female)	11/12	13/13	0.879^b^^†^
Eyes (R/L)	16/7	12/14	0.098^b^
Hyperthyroidism (N/Y)	4/19	4/22	0.850^b^
I131 therapy (N/Y)	17/6	22/4	0.354^b^
Smoking history (N/Y)	14 /9	16/10	0.195^b^
Active phase (N/Y)	14/9	11/15	0.482^b^
CAS	2.13 ± 1.63	3.19 ± 1.86	**0.038**^a^
Duration (m)	5.78 ± 3.46	4.69 ± 1.74	0.182^a^
Exophthalmos (mm)	19.22 ± 3.72	19.35 ± 2.83	0.491^a^
IOP (mmHg)	18.80 ± 5.92	18.94 ± 3.84	0.919^a^
SE (D)	−0.46 ± 1.42	−0.20 ± 1.10	0.491^a^
BCVA	0.97 ± 0.12	0.66 ± 0.23	**<0.0001**^a^
Colour vision abnormal (N/Y)	23/0	25/1	1.000^c^
RAPD (N/Y)	23 /0	29/2	0.492^c^
Decreased VA (N/Y)	23/0	10/16	**<0.0001**^c^
Papilledema (N/Y)	23/0	20/6	**0.0237**^c^
VF defect (N/Y)	6/17	0 /25	**0.008**^c^
P-VEP abnormal (N/Y)	23/0	7/19	**<0.0001**^c^

*DON* dysthyroid optic neuropathy, *CAS* clinical activation score, *IOP* intraocular pressure, *SE* spherical equivalent, *BCVA* best-corrected visual acuity, *VF* visual field, *p-VEP* pattern visual evoked potentials, *RAPD* relative afferent pupillary defect.

^a^Mann–Whitney *U*-test.

^b^Pearson’s Chi-square test.

^c^Fisher’s exact test. Data are mean ± standard deviation or values, with statistically significant *p* values in bold.

### The parameters of icVEP, pVEP, and SAP of the subjects

The results of icVEP, pVEP, and SAP tests in DON and non-DON eyes are summarised in Table 2. In icVEP testing, SNR values in DON group were significantly smaller than those of non-DON group under 8% (*p* < 0.0001), 16% (*p* = 0.0015), and 32% DOM (*p* = 0.0033). In pVEP testing, the value of P100 latent time in DON group was significantly larger than that of non-DON group (*p* = 0.0026), whereas the P100 amplitude of DON was smaller than that of non-DON, though this difference was not significant (*p* = 0.4309). In SAP testing, the value of PSD in DON was larger than that in non-DON (*p* = 0.0006), and the value of MD in DON was smaller than that in non-DON (*p* = 0.0007).

**Table 2:** The parameters of icVEP, pVEP, and SAP of the subjects.

Test	Non-DON (*n* = 23)	DON (*n* = 26)	*p* value
SNR (8%DOM)	1.56 ± 0.68	0.84 ± 0.41	<0.0001*
SNR (16%DOM)	1.81 ± 0.95	0.98 ± 0.74	0.0015*
SNR (32%DOM)	2.20 ± 1.54	1.10 ± 0.62	0.0033*
P100 latent time (ms)	104.48 ± 5.30	114.96 ± 15.01	0.0026*
P100 amplitude (mv)	9.45 ± 5.74	8.21 ± 5.05	0.4309
PSD (VF)	2.23 ± 1.41	4.83 ± 3.09	0.0006*
MD (VF)	−1.82 ± 1.30	−5.58 ± 4.73	0.0007*

All the statistical analysis in the table was using Mann–Whitney *U-*test.

*PSD* pattern standard deviation, *MD* mean deviation, *SNR* signal-to-noise ratio, *DOM* depth of modulation of the check luminance, *DON* dysthyroid optic neuropathy.

*p < 0.05.

### ROC curve analysis for icVEP, pVEP, and SAP

**Fig. 1:**
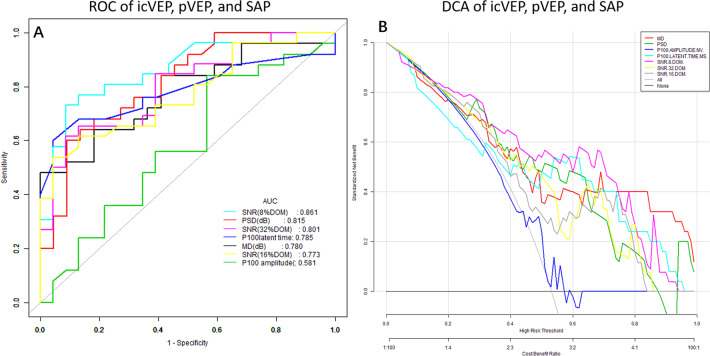
ROC and DCA of icVEP, pVEP, and SAP. **A** ROC of icVEP, pVEP, and SAP showed that the AUC of the SNR in the icVEP test under 8% DOM was the largest, at 0.861, followed by those of PSD, SNR under 32% DOM, P100 latent time, MD, SNR under 16% DOM, and lastly P100 amplitude; **B** DCA of icVEP, pVEP, and SAP showed that the purple curve (8% DOM) is furthest from the two extreme curves, demonstrating that subjects would clinically benefit most from the application of this diagnostic parameter.

The ROC curve analysis for icVEP, pVEP, and SAP parameters is presented in Fig. 1A. The AUC of SNR in icVEP test under 8% DOM was the largest, at 0.861 (95% CI: 0.756–0.966), followed by those of PSD (0.815, 95% CI: 0.694–0.937), SNR under 32% DOM (0.801, 95% CI: 0.678–0.924), P100 latent time (0.785, 95% CI: 0.648–0.923), MD (0.780, 95% CI: 0.647–0.913), SNR under 16% DOM (0.773, 95% CI: 0.643–0.904), and lastly P100 amplitude (0.581, 95% CI: 0.414–0.747). The diagnostic accuracy of the SNR under 8% DOM (81.63%) was higher than the others, while P100 amplitude had the highest sensitivity (84%). SNRs of icVEP under 8 and 32% DOM both had a specificity of 91.30%. SNR under 16% DOM and P100 latent time both had a specificity of 95.65%. The specificity of PSD was 90.91%. MD had the largest specificity (100%), and P100 amplitude had the smallest (43.48%). All of the above are summarised in Table 3.

**Table 3:** ROC parameters of icVEP, pVEP, and SAP.

Test	AUC	95% CI	Best threshold	Specificity	Sensitivity	Accuracy	P-pv	N-pv
MD (dB)	0.780	0.647–0.913	−4.275	1.000	0.480	0.723	1.000	0.629
PSD (dB)	0.815	0.694–0.937	3.660	0.909	0.600	0.745	0.882	0.667
P100 amplitude (mv)	0.581	0.414–0.747	11.350	0.435	0.840	0.646	0.618	0.714
P100 latent time (ms)	0.785	0.648–0.923	113.000	0.957	0.600	0.771	0.938	0.688
SNR (8% DOM)	0.861	0.756–0.966	0.910	0.913	0.731	0.816	0.905	0.750
SNR (16% DOM)	0.773	0.643–0.904	0.805	0.957	0.539	0.755	0.889	0.677
SNR (32% DOM)	0.801	0.678–0.924	1.120	0.913	0.615	0.7347	0.933	0.647

*PSD* pattern standard deviation, *MD* mean deviation, *SNR* signal-to-noise ratio, *DOM* depth of modulation of the check luminance, *ROC* receiver operating characteristic, *AUC* area under the curve, *95% CI* 95% confidence interval, *P-pv* positive predictive value, *N-pv* negative predictive value.

### Discriminative ability of icVEP, pVEP, and SAP

The discriminative ability of icVEP, pVEP, and SAP are analysed in Table 4. We selected the parameter with the largest AUC value in each of the three tests for comparison: SNR under 8% DOM in icVEP, P100 latent time in pVEP, and PSD in SAP. We also calculated the NRI and IDI of those parameters for further analysis. We found that the AUC (8% DOM vs PSD, *p* = 0.664 > 0.05; 8% DOM vs P100 latent time, *p* = 0.434 > 0.05; PSD vs P100 latent time, *p* = 0.590 > 0.05), NRI (8% DOM vs PSD, −0.120, *p* = 0.477 > 0.05; 8% DOM vs P100 latent time, −0.077, *p* = 0.502 > 0.05; PSD vs P100 latent time, 0.004, *p* = 0.975 > 0.05), and IDI (8% DOM vs PSD, −0.120, *p* = 0.486 > 0.05; 8% DOM vs P100 latent time, −0.077, *p* = 0.511 > 0.05; PSD vs P100 latent time, 0.004, *p* = 0.975 > 0.05) among three presented no statistically difference, which indicates that icVEP, pVEP, and SAP showed no significant difference in discriminating DON and non-DON.

**Table 4:** NRI and IDI of icVEP, pVEP, and SAP.

Test	AUC	P1	NRI	P2	IDI	P3
SNR (8% DOM) vs PSD	0.861 vs 0.815	0.664	−0.120	0.477	−0.120	0.486
SNR (8% DOM) vs P100 latent time	0.861 vs 0.785	0.434	−0.077	0.502	−0.077	0.511
PSD vs P100 latent time	0.815 vs 0.785	0.590	0.004	0.975	0.004	0.975

*SNR* signal-to-noise ratio, *DOM* depth of modulation of the check luminance, *AUC* area under the curve, *NRI* net reclassification index, *IDI* integrated discrimination index.

### Clinical utility of icVEP, pVEP, and SAP

We applied DCA to the above three tests to diagnose DON from a clinical-benefit perspective. The net benefit (NB) curves are shown in Fig. 1B. The diagonal light-grey solid line represents all subjects having the outcome (that is, DON), while the horizontal dark-grey solid line represents all subjects not having the outcome (that is, non-DON). These two lines represent two extreme cases. The abscissa is the threshold rate, and the ordinate indicates the NB after pros and cons. The preferred parameter is the one with the highest NB at any given threshold. Thus, according to Fig. 1B, the purple curve (8% DOM) is furthest from the two extreme curves, demonstrating that subjects would clinically benefit most from the application of this diagnostic parameter.

## Discussion

Clinical ophthalmic examinations as well as ancillary tests are attempts to assess the presence, predict the development and estimate the severity of DON. It has been shown that the clinical diagnosis of DON is based on a constellation of findings, including decreased VA, RAPD, colour visual defect, papilledema, VF defects, abnormal pVEP, and evidence of apical crowding on radiographic imaging [3]; however, symptoms, such as decreased VA and defective colour vision are easily affected by patients’ subjective situation and sometimes confused by opaque ocular media. Signs, such as RAPD and papilledema are often not synchronised with the development of disease, resulting in delayed diagnosis. Furthermore, the strength of radiographic signals varies with time and between machines [13] and does not always provide strong evidence of a correlation with DON. Leave VF tests and VEPs to help tailor the prediction of functional abnormalities in the visual system of DON prior to structural changes, and the objectivity and sensitivity of these tests were the other reasons we chose them for comparison.

VEPs are the electrophysiological signals extracted from visual cortex during visual stimulation over the whole system, and any disturbance along the visual pathway results in VEP abnormalities (decrease in amplitude or increase in latency), suggesting the presence of optic neuropathy [6]. However, pVEP test assesses the absolute value of cortical potentials evoked by patterned stimuli [14], which are prone to fluctuate and are affected by the interference of external noise. More particularly, pVEPs are mainly dominated by the central 10° of the VF and will miss localised changes if they are in the periphery [15], which could be compensated by VF testing (e.g., SAP). Such tests detect visual loss in the periphery [16], but they still have disadvantages. First, they suffer from learning effects and are relatively time-consuming, which might easily cause subject fatigue, thus affecting the results. Second, SAP results are not only affected by the opacity of refractive media but easily disturbed by subjective consciousness. Third, studies [17] have reported that SAP is prone to miss damage in the central 10° region, leading to an incomplete result. Last but not least, increased thyroid hormones stimulate sympathetic nervous excitement, with clinical manifestations of palpitation, insomnia, emotional agitation, or even anxiety, accompanied by tremoring [18]. All of the above will greatly influence the result, making it a less-than-perfect parameter for detecting DON. In spite of these, the above two have become the classic methods of functional assessment of optic neuropathy in TAO.

icVEP is a novel technique that offers an objective measurement of visual functions in magnocellular (M-) from parvocellular (P-) pathways. In humans, M cells have relatively large-diameter axons that could be preferentially damaged in optic neuropathy [19, 20], and this specific characteristic of icVEP enables it to detect the earliest changes in visual system. In addition, icVEP records the ratio of signal to noise, a relative value, which can better exclude the influence of external interference than pVEP, thus making the results more objective and comparative among multiple series tests, and making it more suitable for the long-term follow-up of subjects. Therefore, we ran the above three tests to explore their ability to diagnose DON from a more comprehensive methodological perspective: discrimination and clinical utility.

### Comparison of discrimination among icVEP, pVEP, and SAP

Due to its limited availability and inherent variability, several studies have shown that an increase in P100 latency [6, 12, 21] and a decrease in P100 amplitude [6, 12] of pVEP in eyes with DON compared with non-DON were promising for the diagnosis and monitoring of the disease, which is also consistent with our results. However, our study showed that although P100 amplitude of pVEP in DON was smaller than that in non-DON, there was no significant difference between the two. A similar situation was found in ROC curve analysis, in which P100 amplitude had the smallest AUC, indicating that this parameter might contribute least in discriminating DON from non-DON. The discrepancy between our study and the former study might be due to the smaller sample size or the slight age imbalance.

According to our results, a significant increase in PSD and decrease in MD in SAP were found in DON compared with non-DON, which is consistent with similar studies [16, 22, 23] based on optic neuropathy in glaucoma. Although there is no any study specifically on SAP of DON compared with non-DON to our knowledge, it is understandable that chronic DON may cause structural changes at the retinal level [24], thus leading to the changes on SAP.

The SNR of icVEP under different contrast conditions in DON group was significantly lower than that in non-DON. Although no study has analysed the diagnostic power of icVEP in DON patients, our results suggest that icVEP may be an efficient test for predicting DON. Meanwhile, according to ROC curve analysis, SNR under 8% DOM had the largest AUC (0.861), which further suggests that icVEP may be able to detect optic neuropathy earlier than other tests due to its ability to independently monitor changes in the M-pathway.

Together, the significant differences of the three detections between two groups demonstrate that all three tests could be used to distinguish DON from non-DON. Although the AUC values of each parameter varied from high to low, there was no statistical significance among them, indicating that icVEP, pVEP, and SAP share the same discriminative ability for diagnosing DON.

To overcome the deficiencies of the AUC, as well as to further assess and improve sensitivity and specificity [25], we applied NRI and IDI for analysis. According to our results, SNR of icVEP under 8% DOM outperformed both PSD of SAPs and P100 latent time of pVEPs, while PSD outperformed P100 latent time; however, all the comparisons above were without significant differences, illustrating that icVEP, pVEP, and SAP are equally good at predicting DON.

### Clinical utility of icVEP, pVEP, and SAP

Although a test with better discrimination should theoretically function as a better guide to clinical management, statistical measures, such as ROC curve analyses, fall short when we want to evaluate whether the test has clinical utility and improves clinical decision making [26]. Given the same discrimination of the three tests in our study, we applied DCA to further test their clinical utility. We found that 8% DOM in icVEP had a higher NB for the range of reasonable thresholds; thus, it has relatively more clinical utility than the others, and patients might benefit better from undergoing icVEP under 8% DOM than the other two during diagnosis.

When healthcare workers apply a diagnostic test on patients, it is important to consider not only the accuracy and discrimination of the test but the patient’s compliance, cooperation, and financial means. With the whole process completed in less than 2 min and better clinical benefit for patients, icVEP may represent a significant ancillary diagnostic approach to DON detection.

Our study had several limitations. We included 49 eyes from 49 subjects, and a larger sample size needs to be explored for further analysis. However, patients with DON are relatively hard to find because TAO is currently under better control, and fewer patients are going to progress to a severe stage. To our knowledge, this is the largest sample of DON in the field of electrophysiology. In addition, there was a slight age imbalance between the two groups, as patients with non-DON were younger than patients with DON. However, studies have revealed that DON typically develops in an older population [7, 11, 27]; thus, a larger sample size may also compensate for this limitation.

## Conclusion

Our study is the first to analyse and compare the diagnostic ability of icVEP, pVEP, and SAP on DON. We found that these three tests have equal diagnostic capabilities to discern between DON and non-DON. In addition, icVEP may represent a good ancillary diagnostic approach to DON detection with better clinical benefit.

## Summary

- We aim to determine the diagnostic ability of isolated-check visual evoked potential (icVEP), pattern visual evoked potential (pVEP), and standardautomated perimetry (SAP) between dysthyroid optic neuropathy (DON) and thyroid-associated ophthalmopathy (TAO) without DON (non-DON). According to our study, icVEP, pVEP, and SAP have equal diagnostic capabilities to discern between DON and non-DON. Additionally, icVEP mayrepresent a significant ancillary diagnostic approach to DON detection, with more clinical benefit.

## Data sharing

The data collected for the study are available to the publication.

## Data Availability

The data supporting the conclusions of the article are included within the article and its files.

## References

[1] Wong Y, Dickinson J, Perros P, Dayan C, Veeramani P, Morris D (2018). A British Ophthalmological Surveillance Unit (BOSU) study into dysthyroid optic neuropathy in the United Kingdom. Eye.

[2] McKeag D, Lane C, Lazarus JH, Baldeschi L, Boboridis K, Dickinson AJ (2007). Clinical features of dysthyroid optic neuropathy: a European Group on Graves’ Orbitopathy (EUGOGO) survey. Br J Ophthalmol.

[3] Saeed P, Tavakoli Rad S, Bisschop P (2018). Dysthyroid optic neuropathy. Ophthalmic Plast Reconstr Surg.

[4] Bahn RS (2010). Graves’ Ophthalmopathy. N. Engl J Med.

[5] Bartalena L, Baldeschi L, Boboridis K, Eckstein A, Kahaly GJ, Marcocci C (2016). The 2016 European Thyroid Association/European Group on Graves’ Orbitopathy Guidelines for the management of graves’ orbitopathy. Eur Thyroid J.

[6] Iao TWU, Rong SS, Ling AN, Brelén ME, Young AL, Chong KKL (2017). Electrophysiological studies in thyroid associated orbitopathy: a systematic review. Sci Rep.

[7] Blandford AD, Zhang D, Chundury RV, Perry JD (2017). Dysthyroid optic neuropathy: update on pathogenesis, diagnosis, and management. Expert Rev Ophthalmol.

[8] Amarasekera DC, Resende AF, Waisbourd M, Puri S, Moster MR, Hark LA (2018). Steady-state pattern electroretinogram and short-duration transient visual evoked potentials in glaucomatous and healthy eyes. Clin Exp Ophthalmol.

[9] Chen X, Zhao Y (2017). Diagnostic performance of isolated-check visual evoked potential versus retinal ganglion cell-inner plexiform layer analysis in early primary open-angle glaucoma. BMC Ophthalmol.

[10] Xu LJ, Zhang L, Li SL, Zemon V, Virgili G, Liang YB (2017). Accuracy of isolated-check visual evoked potential technique for diagnosing primary open-angle glaucoma. Doc Ophthalmologica.

[11] Dolman PJ (2018). Grading severity and activity in thyroid eye disease. Ophthalmic Plast Reconstr Surg.

[12] Tsaloumas MD, Good PA, Burdon MA, Misson GP (1994). Flash and Pattern visual evoked potentials in the diagnosis and monitoring of dysthyroid optic neuropathy. Eye.

[13] Roos JCP, Murthy R (2019). Update on the clinical assessment and management of thyroid eye disease. Curr Opin Ophthalmol.

[14] Parisi V, Miglior S, Manni G, Centofanti M, Bucci MG (2006). Clinical ability of pattern electroretinograms and visual evoked potentials in detecting visual dysfunction in ocular hypertension and glaucoma. Ophthalmology.

[15] Perez-Rico C, Rodriguez-Gonzalez N, Arevalo-Serrano J, Blanco R (2012). Evaluation of multifocal visual evoked potentials in patients with Graves’ orbitopathy and subclinical optic nerve involvement. Doc Ophthalmol.

[16] Fortune B, Demirel S, Zhang X, Hood DC, Patterson E, Jamil A (2007). Comparing multifocal VEP and standard automated perimetry in high-risk ocular hypertension and early glaucoma. Invest Ophthalmol Vis Sci.

[17] Park HL, Lee J, Park CK (2018). Visual field tests for glaucoma patients with initial macular damage: comparison between frequency-doubling technology and standard automated perimetry using 24-2 or 10-2 visual fields. J Glaucoma.

[18] Ross DS, Burch HB, Cooper DS, Greenlee MC, Laurberg P, Maia AL (2016). 2016 American thyroid association guidelines for diagnosis and management of hyperthyroidism and other causes of thyrotoxicosis. Thyroid.

[19] Liu Z, Chen Z, Xu Y, Feng L, Yuan J, Deng D (2020). Objective assessment of the effect of optical treatment on magnocellular and parvocellular-biased visual response in anisometropic amblyopia. Invest Ophthalmol Vis Sci.

[20] Fan X, Wu LL, Di X, Ding T, Ding AH (2018). Applications of isolated-check visual evoked potential in early stage of open-angle glaucoma patients. Chin Med J (Engl).

[21] Debniak AR, Lubinski W, Krzystolik Z (1999). Visual evoked potentials in diagnosis and monitoring of optic neuropathy in the course of thyroid ophthalmopathy. Klin Ocz.

[22] Hirasawa K, Takahashi N, Satou T, Kasahara M, Matsumura K, Shoji N (2017). Comparison of Size modulation standard automated perimetry and conventional standard automated perimetry with a 10-2 test program in glaucoma patients. Curr Eye Res.

[23] Takahashi G, Demirel S, Johnson CA (2017). Predicting conversion to glaucoma using standard automated perimetry and frequency doubling technology. Graefes Arch Clin Exp Ophthalmol.

[24] Park KA, Kim YD, In Woo K, Kee C, Han JC (2016). Optical coherence tomography measurements in compressive optic neuropathy associated with dysthyroid orbitopathy. Graefes Arch Clin Exp Ophthalmol.

[25] Pencina MJ, Sr RBDA, Jr PBDA, Vasan RS (2008). Evaluating the added predictive ability of a new marker: From area under the ROC curve to reclassification and beyond. Stat Med.

[26] Van Calster Ben, Wynants Laure, Verbeek JanFM, Verbakel JanY, Christodoulou Evangelia, Vickers AndrewJ (2018). Reporting and interpreting decision curve analysis: a guide for investigators. Eur Urol.

[27] Ugradar S, Rootman DB (2019). Noninflammatory thyroid eye disease. Ophthalmic Plast reconstructive Surg.

